# Adaptive Enhancement of X-Band Marine Radar Imagery to Detect Oil Spill Segments

**DOI:** 10.3390/s17102349

**Published:** 2017-10-14

**Authors:** Peng Liu, Ying Li, Jin Xu, Xueyuan Zhu

**Affiliations:** Environmental Information Institute of Navigation College, Dalian Maritime University, Dalian 116026, China; liupeng@dlmu.edu.cn (P.L.); xujin_1985@126.com (J.X.); zhuxueyuan1206@163.com (X.Z.)

**Keywords:** X-band marine radar, oil spill detection, adaptive algorithm

## Abstract

Oil spills generate a large cost in environmental and economic terms. Their identification plays an important role in oil-spill response. We propose an oil spill detection method with improved adaptive enhancement on X-band marine radar systems. The radar images used in this paper were acquired on 21 July 2010, from the teaching-training ship “YUKUN” of the Dalian Maritime University. According to the shape characteristic of co-channel interference, two convolutional filters are used to detect the location of the interference, followed by a mean filter to erase the interference. Small objects, such as bright speckles, are taken as a mask in the radar image and improved by the Fields-of-Experts model. The region marked by strong reflected signals from the sea’s surface is selected to identify oil spills. The selected region is subject to improved adaptive enhancement designed based on features of radar images. With the proposed adaptive enhancement technique, calculated oil spill detection is comparable to visual interpretation in accuracy.

## 1. Introduction

Oil spills are one of the major environmental hazards in ocean basins. According to studies of Alves et al. in the Mediterranean Basin [1,2,3,4,5], in oil spill accidents, crude oil at, and beneath, the water’s surface can quickly spread and reach the coastline under particular weather and oceanographic conditions. Hence, prompt and accurate oil spill detection is a critical step in any contingency plan. Marine radars, which are used to detect oil spills [6], are highly accessible on vessels. *International Convention for the Safety of Life at Sea* [7] stipulates that ships of 500 gross tonnage and upwards constructed on or after 1 September 1984, and ships of 1600 gross tonnage and upwards constructed before 1 September 1984, shall be fitted with a radar installation. Therefore, for our purpose, no additional radars have to be purchased and the radar images are available on the navigation radar. Compared with other oil spill detection methods, such as laser fluorescence [8], optical sensors [9], and SAR [10], which call for specialized devices or expensive satellite images in the contingency plan, marine radars are more convenient, expedient, and economical. In marine radar images, the backscattered signal intensity of an oil spill area is weaker than that of the neighboring waters. This contrast can be exploited to tease apart oil spills [11]. Some commercial systems using marine radars have been developed, including the oil spill detection (OSD) system of Miros (Asker, Norway) [12,13] and the SeaDarQ radar system of Nortek B.V. (Badhoevedorp, Netherlands) [14,15]. However, due to commercial competition, the identification methods are seldom publicized. Scholarly studies in this area are also sparse and outdated [6,16].

Zhu et al. developed a simple OSD method [17] to access oil spill information. In their study, over 30 radar images were used to map and predict the attenuation of signal intensity in radar images taken at varied distances. According to the overall estimated attenuation of signal intensity, the individual radar images were modified and a manually-set global threshold was applied to visualize the oil spills. One of the limitations in this method is that it requires an extended period of time and a large number of images to calculate the attenuation of the signal intensity, which is also affected by the ship’s location and direction. Another drawback lies in its overall estimated attenuation of signal intensity. Its overall estimated result cannot fully reflect the signal intensity in individually-measured radar images. Consequently, a significant error of oil spill visualization arises.

In this paper, we propose an improved adaptive enhancement for detecting oil spills based on X-band marine radar images. Section 2 will lay out how radar images were obtained and preprocessed by erasing co-channel interference, clearing small object turbulence and narrowing down operational regions. Section 3 explores an improved algorithm to visualize oil spills. Radar images are broken into thin strips and the Otus method is used to detect oil spill segments in each strip. Since different strip thicknesses render different segmentation results, the final oil spill area is obtained based on the number of oil spill appearances broken down to various extents. In Section 4, the proposed improved adaptive enhancement will be compared with methods proposed in Bradley et al. and Zhu et al. Section 5 is the conclusion.

## 2. Data Collecting and Preprocessing

### 2.1. Radar Image Collection

**Figure 1:**
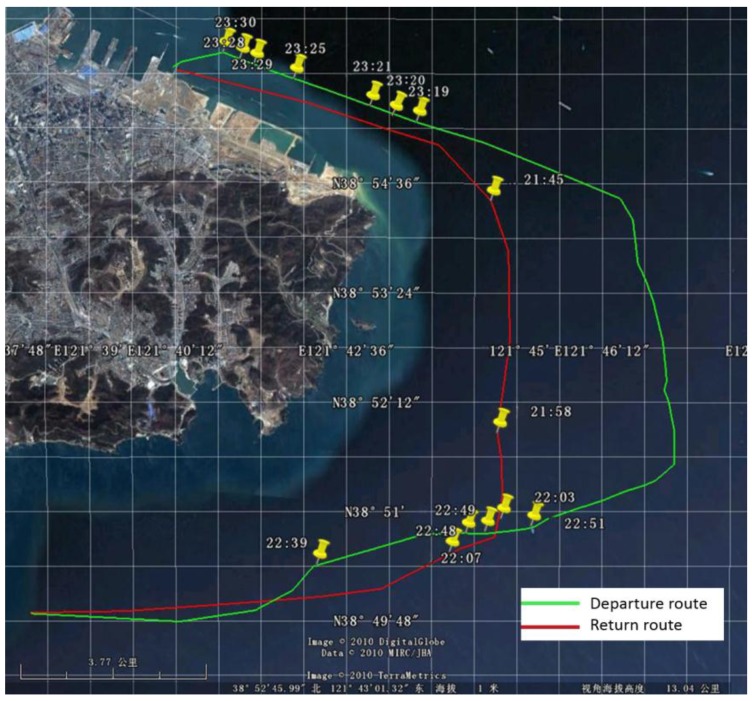
Navigation route of the teaching-training ship “YUKUN” on 21 July 2010.

**Figure 2:**
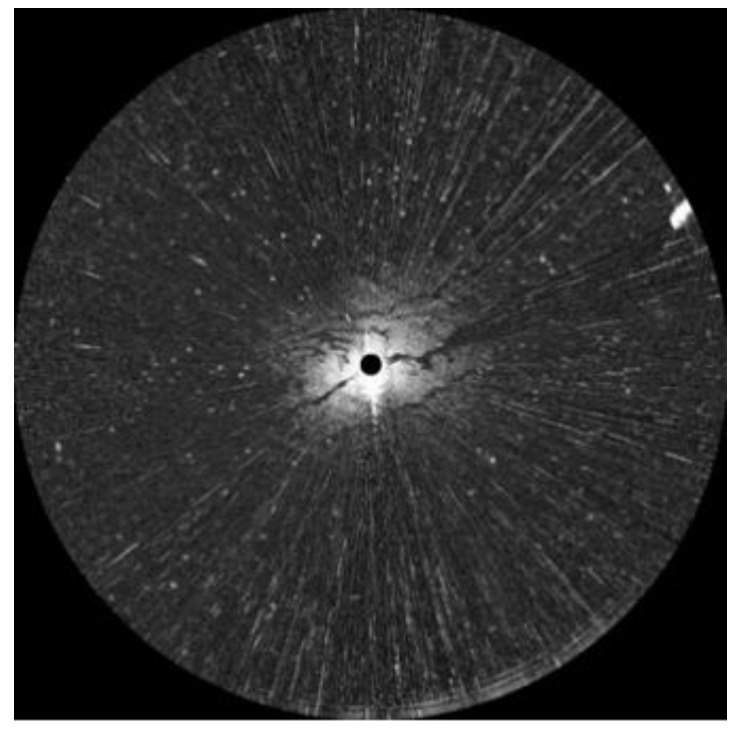
Original radar image.

**Figure 3:**
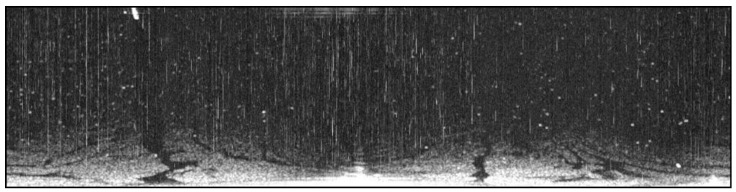
Converted radar image.

An oil spill accident took place in Dalian on 16 July 2010. The teaching-training ship “YUKUN”, owned by Dalian Maritime University, was used to sample the oil spill on 21 July, and the route is shown in Figure 1. An X-band marine radar installed on “YUKUN” was used. The radar used was from Sperry Marine (Head Office in London, UK) and its main parameters are shown in Table 1. One example of radar image with a scanning radius of 0.75 nautical miles at 23:19, 21 July 2010, is shown in Figure 2. The marine radar images were plotted on a Cartesian coordinate system instead of the polar coordinate system for more straightforward analysis. An example is shown in Figure 3, the image resolution being 512 × 2048. In Figure 3, the horizontal axis indicates angles of incidence, the vertical axis distances from the pole and the brightness the strength of reflected signal.

### 2.2. Radar Image Preprocessing

#### 2.2.1. Co-Channel Interference

**Figure 4:**
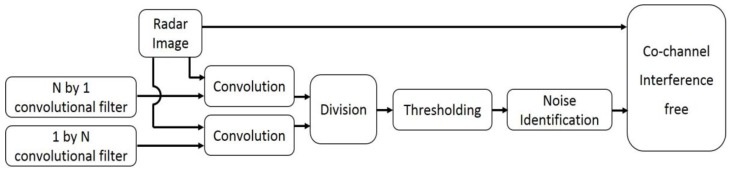
Data processing of clearing co-channel interference.

As shown in Figure 3, co-channel interference manifests as bright band effects, preventing us from accessing the desired information. Therefore, erasing co-channel interference is prioritized in processing radar images. The appearance of co-channel interference shows that the signal intensity is much larger in its vertical direction than in its horizontal direction. Based on this feature, the effects of two convolutional filters, a 1 × *N* matrix and an *N* × 1 matrix, were divided to detect the co-channel interference and a mean filter was applied to erase it. The detecting and erasing of co-channel interference is shown in Figure 4. An index used to identify the co-channel interference is expressed as:

(1) $$C\left(i,j\right)=\frac{\sum_{k=-\left(N-1\right)/2}^{\left(N-1\right)/2}I\left(i,j+k\right)V_{c}\left(k\right)}{\sum_{l=-\left(N-1\right)/2}^{\left(N-1\right)/2}I\left(i+l,j\right)V_{r}\left(l\right)},$$

where *C(i,j)* is the index value; $V_{c}$ is the *N* × 1 convolutional filter; $V_{r}$ is the 1 × *N* convolutional filter; and N is the length of convolutional filter, which is set as an odd number.

For convenience, the value of the index is normalized in the form of integers from 0 to 255. According to the index value, thresholds determined by the Otsu method [18,19] were used to identify the co-channel interference. Then, co-channel interference in identified loci was erased by a mean filter [20].

**Figure 5:**
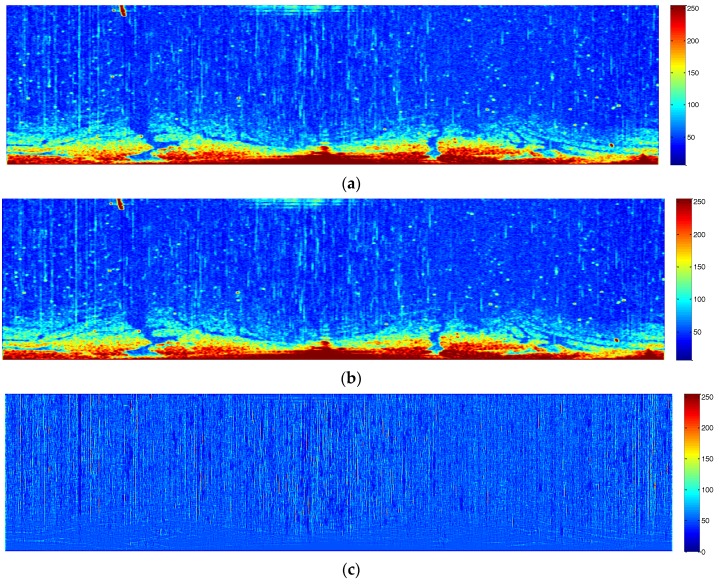

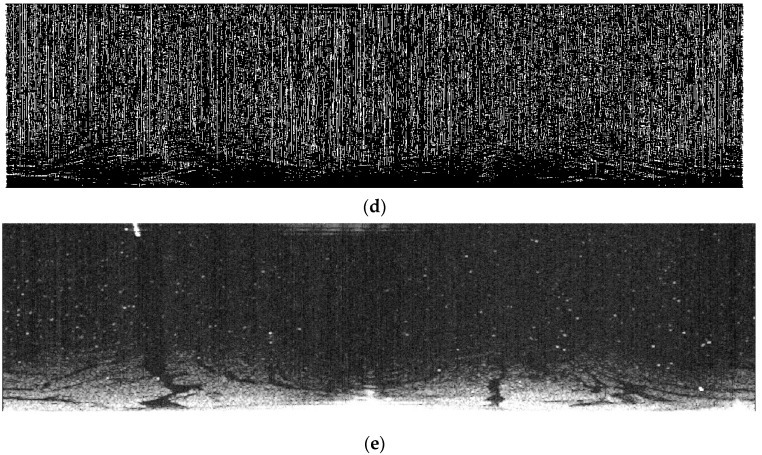
Processed images with co-channel interference removed: (**a**) conventional image with a 7 × 1 filter; (**b**) conventional image with a 1 × 7 filter; (**c**) contrasting image of the two conventional images; (**d**) binarization image with identified co-channel interference; and (**e**) the radar image with co-channel interference removed.

For instance, two convolutional filters were set as [1, 1, 1, 1, 1, 1, 1] and [1; 1; 1; 1; 1; 1; 1], the threshold calculated by the Otsu method was 60, and the window of mean filtering was set as a 1 × 7 matrix. Images free from co-channel interference are shown in Figure 5. Figure 5a,b shows the filtered images by convolutional filters with 7 × 1 and 1 × 7 windows, respectively. Figure 5c shows the index value. Figure 5d is a binarization image with the threshold determined by the Otsu method. Figure 5e is the processed image with co-channel interference erased by a mean filter. Compared with the original sampled image shown in Figure 3, Figure 5e is visibly cleaner and more accurate.

#### 2.2.2. Small Objects

**Figure 6:**
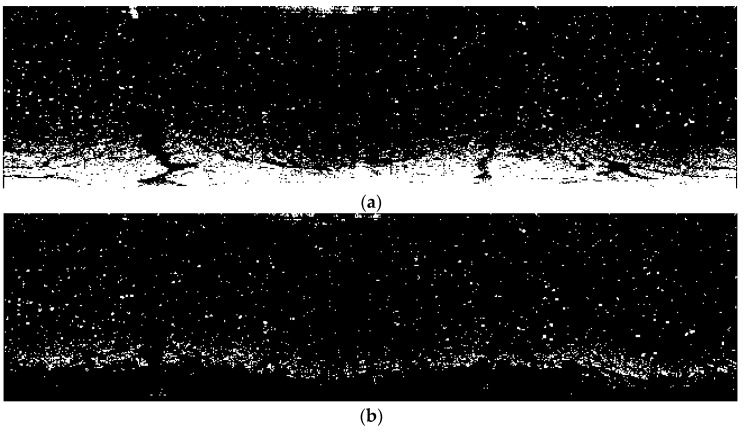

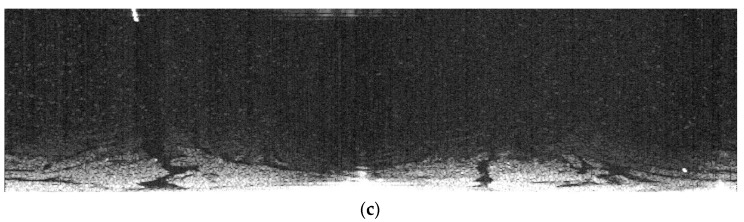
Radar image with small objects erased: (**a**) binarization of the radar image without co-channel interference; (**b**) extracted noise as bright speckles; and (**c**) processed radar image after noise reduction.

Figure 5e, though free from co-channel interference, is still littered with bright speckles, which are caused by reflected signals of small objects. Bright speckles pose a sharp contrast against the background, which affects image quality. Therefore, bright speckles were often considered as noise. Noise reduction was carried out using the Fields-of-Experts (FoE) model [21]. In this method, bright speckles are taken as masks which have to be known as prior information. To gain the information of masks of Figure 5e, binarization with the threshold of 90 was implemented, the result of which is shown in Figure 6a. In addition, all connected components (objects) which have fewer than 200 pixels were extracted as masks, which is shown in Figure 6b. Then, 3 × 3 square patches with eight filters were adopted to clean up the masked image. The processed image is shown in Figure 6c.

#### 2.2.3. Regions Selected for the Current Study

**Figure 7:**
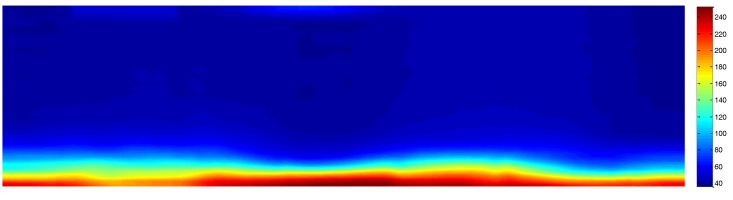
Convolution results of selected regions.

**Figure 8:**
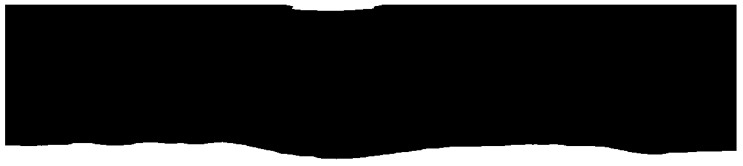
Binarization image of selected regions.

**Figure 9:**
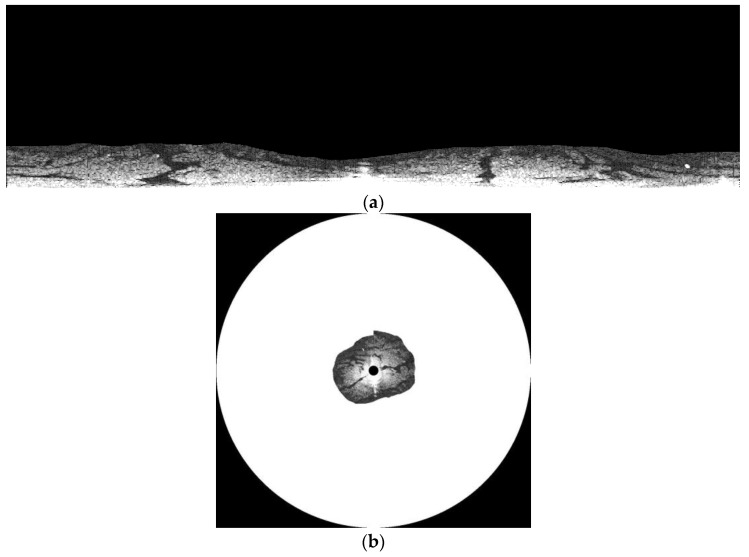
Selected region of radar image for oil spills detection: (**a**) selected region in the Cartesian coordinate system; and (**b**) the selected region in the polar coordinate system.

The intensity of the reflected signal from the sea’s surface obtained by X-band marine radars is affected by distance, roughness of the sea’s surface, and incidence [22,23]. The intensity of the reflected signal is negatively correlated with distance, while the roughness of the sea’s surface contributes to the signal intensity. Signal intensity is also subject to angular changes in the incidence and wave direction. Therefore, the entire the sampled region is not suitable for our study due to poor signal intensity. To pin down the suitable areas, a convolutional filter of a 20 × 160 matrix was used. Figure 7 is the filtered image of Figure 6c, which clearly shows that the prime location for analysis was at the bottom of the image. A threshold of 60 was used to further outline the location, which is shown as a white irregular ribbon in Figure 8. As shown in Figure 8, not only was the region of strong reflected signal from the sea’s surface identified, but the complementary region of strong reflected signals from large objects were also extracted and shown on top of the radar image. However, the sea’s surface far from the radar cannot generate strong reflections, and are unsuitable for our research purposes. The extracted region for oil spill segmentation in our study is shown in Figure 9. Figure 9a,b show the extracted region in the Cartesian coordinate system and the polar coordinate system separately. The area of the selected region is 0.34 km^2^, accounting for 5.61% of the area covered by the radar image.

## 3. Adaptive Enhancement to Detect Oil Spill

As the intensity of the reflected signal from the sea’s surface is affected by distance, roughness of the sea’s surface, and incidence, oil spills cannot be extracted accurately with a one-fit-all threshold. Hence, an adaptive thresholding [24,25,26,27,28] that is normally used in image processing is adopted. The adaptive thresholding proposed by Bradley et al. [29] proved to be an effective tool to extract desirable information in gray images, but it is less powerful in oil spill detection. Considering the features of radar images for oil spill detection, an improved adaptive thresholding is proposed.

**Figure 10:**
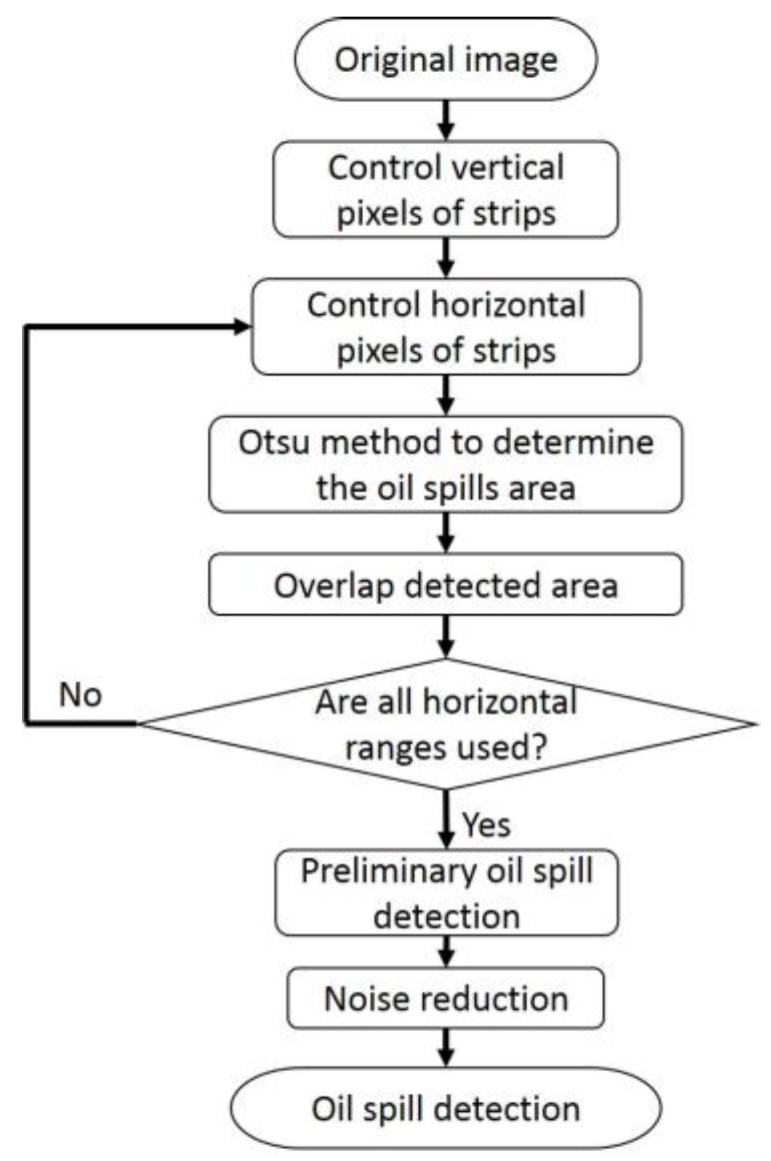
Data processing of the improved adaptive enhancement.

The data processing of proposed method is shown in Figure 10. The radar image was broken down into small strips and an individual threshold was determined for each strip. Since the intensity of radar signals fade quickly with distance, few pixels in the vertical direction of each strip were used. The number of pixels in the horizontal strips varied and generated strips of different thicknesses. With each strip, the Otsu method was adopted to calculate the threshold for oil spills. With different strip thicknesses, different oil spill segments were obtained. Then these extracted oil spills were overlapped. If the number of oil spills appearing in one pixel was larger than half of the strip, this pixel qualifies as a component of an oil spill area. Oil spills often stretch across an uninterrupted region, which means that small extracted oil spills are erroneous and need to be erased. Figure 10 shows how the raw data is processed. A comparison with two other adaptive thresholding methods proposed by Bradley et al. and Zhu et al. will be discussed in the next section.

## 4. Evaluation of the Improved Adaptive Enhancement

**Figure 11:**
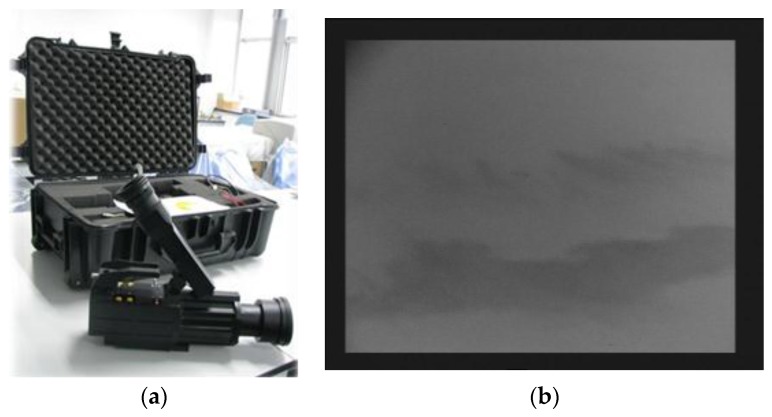
An oil spill image monitored by thermal infrared sensor: (**a**) thermal infrared sensor used in this study; and (**b**) an image captured by thermal infrared sensor.

To exclude possible interferences (e.g., wind turbulence, swells) in the radar image in Figure 2, a ship-borne thermal infrared sensor was used. Provided by Zhejiang Dali Technology Co. Ltd (Hangzhou, China), the sensor has a wavelength ranging from 7.5 to 13 μm. The thermal infrared image captured at 23:19, 21 July 2010 on “YUKUN” is shown in Figure 11. Figure 11b shows that the region covered by the radar contains spilled oil, as opposed to interference.

To evaluate the performance of oil spill detection with our improved adaptive enhancement, a comparison with the adaptive thresholding method proposed by Bradley et al. and the OSD method proposed by Zhu et al. is carried out.

**Figure 12:**
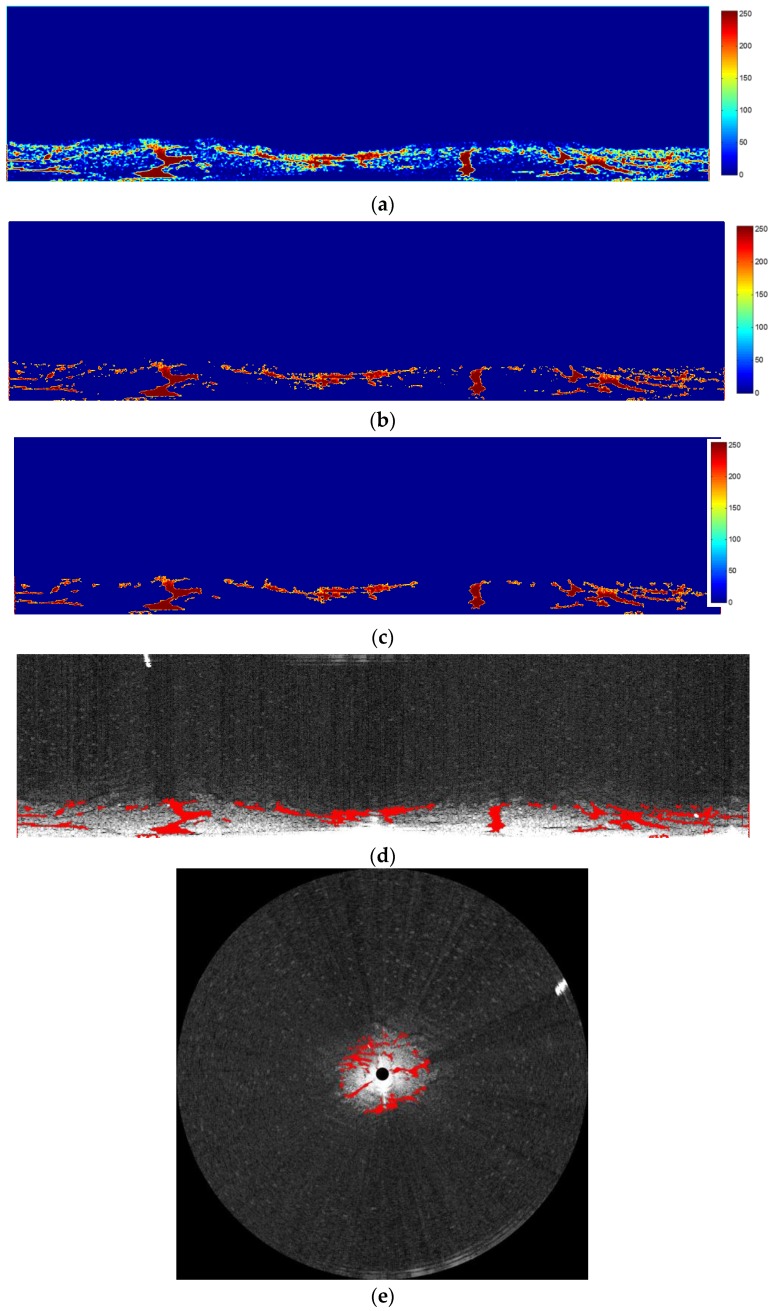
Oil spill segment based on improved adaptive thresholding: (**a**) overlapped image of an oil spill segment with five different strip thicknesses; (**b**) detected oil spill image with noise; (**c**) detected oil spill image with noise removed; (**d**) final processed radar image marked with the detected oil spill in the Cartesian coordinate system; and (**e**) the final processed radar image marked with the detected oil spill in the polar coordinate system.

Before using the improved adaptive thresholding, Figure 2 was first preprocessed as shown in Figure 9. Then the image in Figure 9 was cut into strips of different configurations: 4 × 128, 4 × 256, 4 × 512, 4 × 1024, and 4 × 2048. Based on trhe data processing in Figure 10, the overlapped segments resulted in different strip thicknesses, as shown in Figure 12a. The value of pixels in Figure 12a range from 0 to 255. Then, with the threshold of 128, half of the pixel value range, extracted oil spills are shown in Figure 12b. At this stage, the extracted oil spill still contained noise created by small objects. Figure 12c shows the identified oil spill after all objects with pixels less than 100 were cleared up. In Figure 12d, the identified spill oil is marked on the original radar image in the Cartesian coordinate system. In Figure 12e, the identified spill oil is marked on the original radar image in the polar coordinate system, and the extent of the detected oil spill area is 0.05 km^2^.

**Figure 13:**
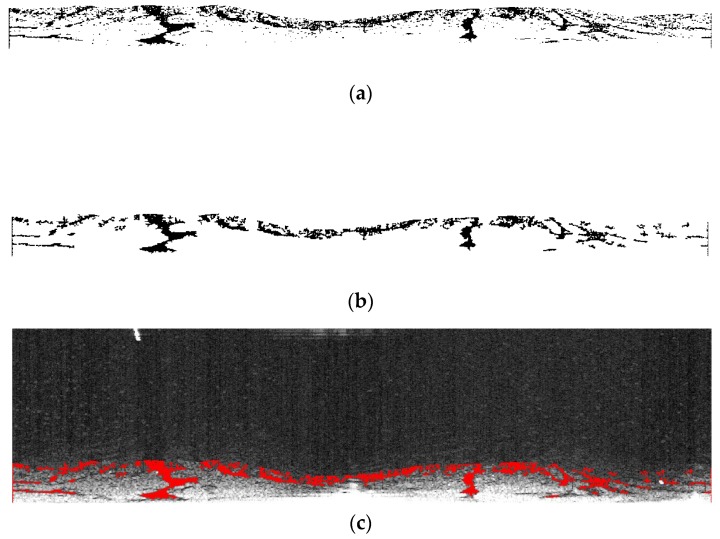
Oil spill segment detected based on adaptive thresholding proposed by Bradley et al.: (**a**) oil spill segment detected by Bradley’s method; (**b**) oil spill segment with noise removed; and (**c**) oil spill segment marked in red on the radar image.

**Figure 14:**
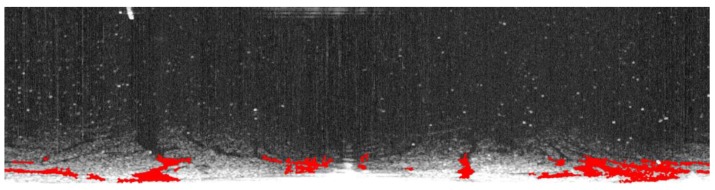
Oil spill segment identified using the OSD method proposed by Zhu et al.

Figure 9 was also processed using the method proposed by Bradley et al. The first step was to calculate the integral image of Figure 9, then an average of 64 × 64 pixels around the current pixel in the integral image of Figure 9 was calculated, and the value of the current pixel was set as 0 if it was 15% less than the average. The detected results based on Bradley’s method are shown in Figure 13a. With small object noise removed, oil spills are shown in Figure 13b and they are marked in red in the original radar image shown in Figure 13c. Zhu et al. used a power attenuation correction method and the extracted oil spill is in red in Figure 14.

**Figure 15:**
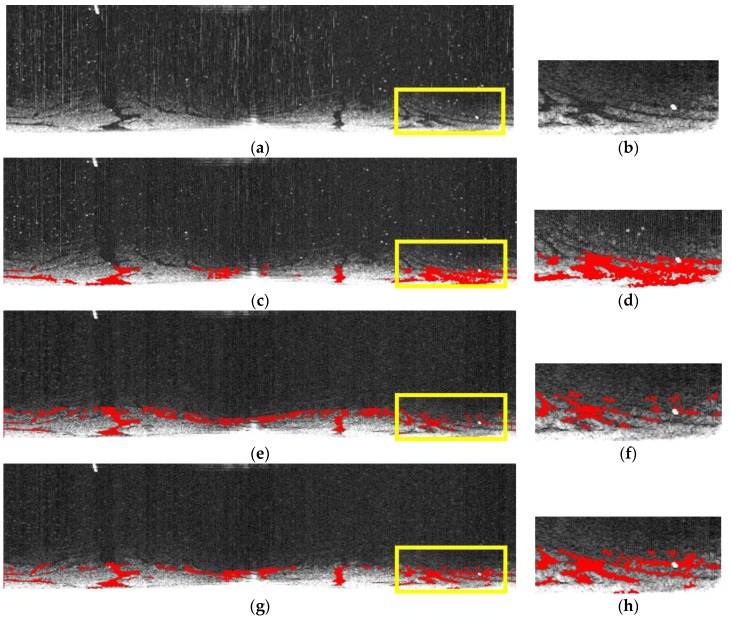
Comparing three methods in extracting oil spills: (**a**) original image; (**b**) enlarged version of the highlighted area in (**a**); (**c**) oil spill detected using the method proposed by Zhu et al.; (**d**) enlarged version of the highlighted area in (**c**); (**e**) oil spill detected using the method proposed by Bradley et al.; (**f**) enlarged version of the highlighted area in (**e**); (**g**) oil spill detected using the improved adaptive enhancement; and (**h**) the enlarged version of the highlighted area in (**g**).

The area picked for comparing three methods contains traces of oil spills in original radar images and oil spill images by Zhu et al., Bradley et al., and our own. The selected areas are amplified and shown in Figure 15b,d,f,h. In Figure 15d, the oil spill area is overrepresented while, in Figure 15f, it is underrepresented. The oil spill area extracted in Figure 15h is the closest match among the three to the naked-eye observation of the original radar image in Figure 15b.

**Figure 16:**
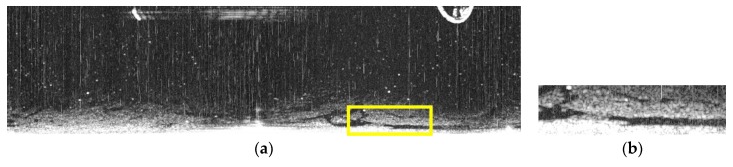

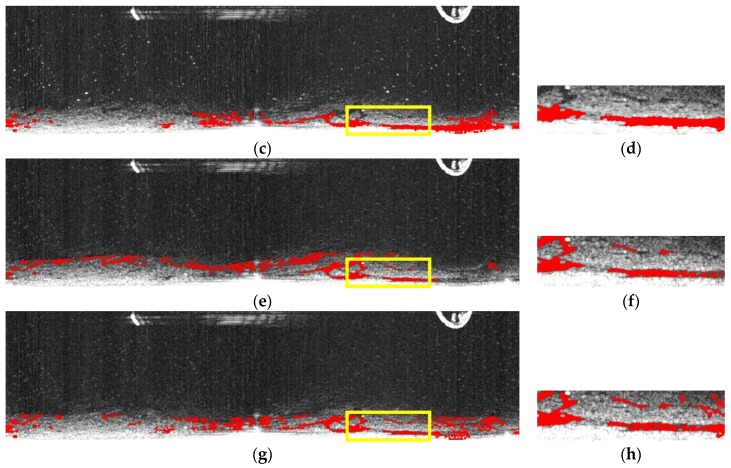
Comparing oil spill detection by three methods: (**a**) original image; (**b**) enlarged version of the highlighted area in (**a**); (**c**) oil spills detected using the method proposed by Zhu et al.; (**d**) enlarged version of the highlighted area in (**c**); (**e**) oil spills detected using the method proposed by Bradley et al.; (**f**) enlarged version of the highlighted area in (**e**); (**g**) oil spills detected using the improved adaptive enhancement; and (**h**) the enlarged version of the highlighted area in (**g**).

**Figure 17:**
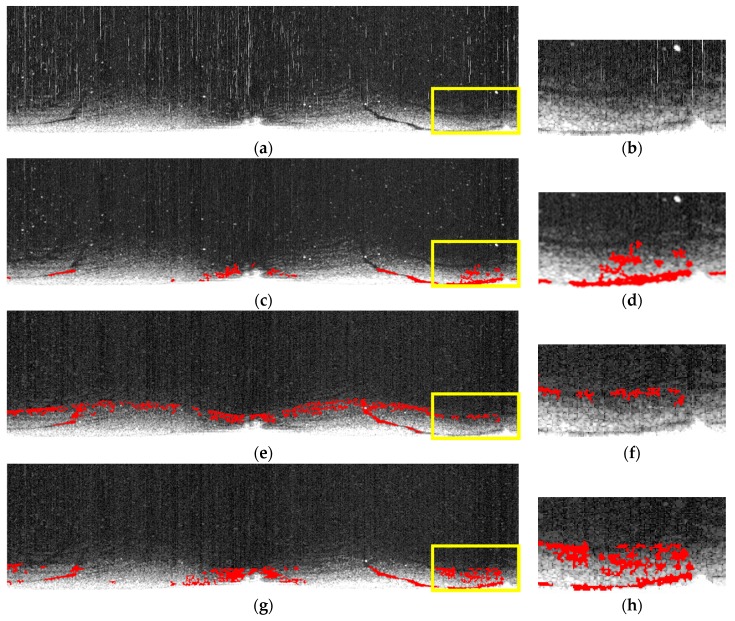
Comparing oil spill detection by three methods: (**a**) original image; (**b**) enlarged version of the highlighted area in (**a**); (**c**) oil spills detected using the method proposed by Zhu et al.; (**d**) enlarged version of the highlighted area in (**c**); (**e**) oil spills detected using the method proposed by Bradley et al.; (**f**) enlarged version of the highlighted area in (**e**); (**g**) oil spills detected using the improved adaptive enhancement; and (**h**) the enlarged version of the highlighted area in (**g**).

Our method proved to be consistently more accurate as shown in another two comparisons in Figure 16 and Figure 17. In Figure 16, the extracted oil spill in the selected area by the method proposed by Zhu et al. (Figure 16d) left out a large oil spill area. Bradley et al. managed to identify more oil spill areas, as shown in Figure 16f, but this method was still unsatisfactory compared to our improved adaptive enhancement method, as shown in Figure 16h. Similarly, in Figure 17, Zhu et al. again lacks accuracy in the upper portion and the method proposed by Bradley et al. misses the oil spills in the lower portion. Our method, in contrast, delineates the oil spill areas containing the most comprehensive information. By comparing three methods on three radar images, it is safe to say that the improved adaptive enhancement based on the characteristics of radar images provides the best oil spill detection solution.

## 5. Conclusions

A new oil spill detection method was proposed using improved adaptive enhancement based on X-band marine radar imagery. First, radar images were preprocessed, including transforming from the polar coordinate system to the Cartesian coordinate system, removing co-channel interference and small object noise, and choosing the suitable target region. Co-channel interference, as per its appearance, was erased using two convolutional filters. Small objects, manifesting as small bright regions, were considered as masks and removed using the FoE model. Before attempting to detect oil spills, the most suitable regions for our research purpose were selected. Then, an improved adaptive thresholding based on the characteristics of radar images was proposed. Radar images were cut into strips of five thicknesses, and the oil spill segments based on different strip thicknesses were overlapped. Oil spill areas were determined with thresholds calculated by the number of appearances of oil spills as per different strip thicknesses. Next, a comparison of the adaptive enhancement technique proposed here and the methods devised by Bradley et al. and Zhu et al. was carried out, where our method is consistently more accurate and comparable to that of the human eye.

## Figures and Tables

**Table 1:** Main parameters of X-band marine radar.

Name		Parameters
Working frequency		9.41 GHz
Antenna type		Waveguide split antenna
Observation range		0.1~5.0 km
Detection angle	Horizontal direction	360°
	Vertical direction	±10°
Pulse width		50 ns/250 ns/750 ns
Pulse Repetition frequency		3000 Hz/1800 Hz/785 Hz
